# 

**Published:** 1858-06

**Authors:** 


### RECEIPTS ON SUBSCRIPTIONS,

*From May 15th to June 14th*, 1858.

| | | | | | |
|---|---|---|---|---|---|
| Dr. M. Davis, | Illinois. | $2 00 | Dr. C. T. Lichtenberger, | Illinois, | $1 00 |
| " W. R. Fox, | " | 2 00 | " P. L. Diefenback, | " | 2 00 |
| " H. Nance, | " | 2 00 | " L. A. Winslow, | " | 2 00 |
| " F. K. Bailey, | " | 2 00 | " Jossey, | " | 6 00 |
| " J. McCann, | " | 2 00 | " J. Blount, | " | 2 00 |
| " H. Noble, | " | 2 00 | " E. Smith, | " | 2 00 |
| " John Walker, | " | 2 00 | " D. E. Foote, | " | 2 00 |
| " J. P. Rawson, | " | 4 00 | " S. Hagar, | " | 2 00 |
| " J. D. Cope, | " | 2 00 | " F. W. Hance, | " | 4 00 |
| " Mergler, | " | 2 00 | " C. M. Richmond, | Indiana, | 2 00 |
| " E. Andrews, | " | 4 00 | " C. D. Watson, | " | 2 00 |
| " J. N. Niglas, | " | 2 00 | " J. Doran, | Iowa, | 2 00 |
| " J. D. Arnold, | " | 2 00 | " James Carson, | " | 1 00 |
| " R. Rouse, | " | 4 00 | " H. A. Yeomans, | Wisconsin, | 2 00 |
| " E. Dickenson, | " | 4 00 | Thos. E. Bond, | Maryland. | 6 00 |
| " [illegible] A. Engle, | " | 2 00 | | | |

